# Miss M'Kellar and the Hospitals

**Published:** 1888-11-10

**Authors:** 


### MISS M'KELLAR AND THE HOSPITALS.

THE late Miss Louisa M'Kellar, of Argyll Lodge, Clapham Park, by her will, which was proved about twelve months ago, gave the sum of £58,000 in trust for her sister, with reversionary benefits to some 45 different Hospitals and Charitable Institutions, and her residuary estate to trustees (Mr. John Jeffryes Oakley, Mr. William Edward Long, and Mr. Francis William Arkcoll), to distribute amongst such Charitable and Benevolent Institutions as they in their absolute discretion might think fit. In the exercise of this discretion those gentlemen have settled a scheme for the distribution of the residuary estate, amounting to some £45,000, and have apportioned the funds at their disposal amongst various Institutions, purposely excluding those which will ultimately become benefited under the will. The following Medical Charities have benefited :—

HOSPITALS.—Guy's Hospital, £2,000; King's College Hospital, £1,000; London Hospital, £250; Middlesex Hospita £1,000; University College Hospital, £1,000; Royal Fre Hospital, £1,000; Great Northern Hospital, £1,000; West London Hospital, £400; East London Hospital (Shadwell), £400; Metropolitan Hospital, £400; Richmond Hospital, £250; Bartholomew's Hospital, £400; Bethlem Hospital, £400; North-Eastern Hospital for Children, £400; Hospital for Women, Soho Square, £250; Queen Charlotte's Lying-in Hospital, £150; Lying-in Hospital, City Road, £150; General Lying-in Hospital, York Road, £100; New Hospital for Women, £100; Chelsea Hospital for Women, £200; Samaritan Free Hospital, £200; National Hospital for Diseases of the Heart and Paralysis, £100; Royal Maternity Hospital, £100; City of London Chest Hospital, Victoria Park, £250; North London Consumption Hospital, £250; Royal Hospital for Diseases of the Chest, £250; French Hospital, £400; German Hospital, £400; Dental Hospital, £100; National Dental Hospital, £100; Temperance Hospital, £150; Poplar Hospital, £150; Hospital for Diseases of the Throat, £150; London Homœopathic Hospital, £150; Lock Hospital, —; St. Mark's Hospital for Fistula, £150; Royal Hospital for Consumption, Ventnor, £200; St. Peter's Hospital for Stone, £250; Seamen's Hospital, £250; Royal Westminster Ophthalmic Hospital, £150; Royal London Ophthalmic Hospital, £150; Royal Orthopædic Hospital, £150; Royal Sea Bathing, Margate, £100; Hospital for Children, Great Ormond Street, £250; Hospital for Incurable Children, Maida Vale, £100; Hospital for Children, Paddington, £250; Evelina Hospital for Children, £250; Alexandra Hospital for Children, £100; Victoria Hospital for Children, Chelsea, £150; Royal Hospital for Children and Women, £150; Belgrave Hospital for Children, £250; Cottage Hospitals (various), £200.

SURGICAL AID.—Provident Surgical Appliance Society, £100; Surgical Aid Appliance Society, £100; City of London Truss Society, £250; National Industrial Home for Crippled Boys, £250; Cripples' Home and Industrial School for Girls, £250; Home for Poor Crippled and Orphan Boys, Moore-street, £100.

DISPENSARIES.—Western General, Marylebone, £150; St. George's and St. James, King Street, Regent Street, £150; Westbourne Provident, £100; Richmond Dispensary, £150; Finsbury Dispensary, £100; Surrey Dispensary, £250; South London Dispensary, £250; St. Paul and St. Barnabas Dispensary, £200.

CONVALESCENT.—Bournemouth National Sanatorium, £250; Metropolitan Convalescent Institution (Walton, £200, Kingston, £200, Bexhill, £200), £600; Mary Wardell's Home for Scarlet Fever, —; Princess Frederica's, East Moulsey, £100; St. Mary's, Broadstairs, £250; St. John's House of Rest, Mentone, £200; St. Andrew's, Folkestone, £100.

NURSING INSTITUTIONS AND OTHERS.—Metropolitan Association of Nurses, £100; St. John's House Nurses, £100; East London Nursing Society, £100; Royal Medical Benevolent College, £100; British Medical Benevolent Fund, £100; London Samaritan Society, £200; Discharged Prisoners' Aid, £200; Society for the Relief of Widows and Orphans of Medical Men, £100; Mrs. Hilton's Crèche, £100; Royal Alfred Seamen's Institution, £150; Home for Englishwomen in Paris, £200; Establishment for Gentlewomen During Temporary Illness, £250; Home for Female Inebriates, £100; St. John's Convalescent Home, Kemptown, £200; People's Palace, Mile End, £350; South London Polytechnic Institutes, £350; Metropolitan Public Gardens Association, £200.

